# Optic Disc Hemorrhage and Lamina Cribrosa Defects in Glaucoma Progression

**DOI:** 10.1038/s41598-017-03828-0

**Published:** 2017-06-14

**Authors:** Hae-Young Lopilly Park, Jiyoung Lee, Younhea Jung, Chan Kee Park

**Affiliations:** Department of Ophthalmology and Visual Science, Seoul St. Mary’s Hospital, College of Medicine, The Catholic University of Korea, Seoul, Korea

## Abstract

Both disc hemorrhages (DH) and focal lamina cribrosa (LC) defects are recently considered as a progression factor in glaucoma. However, the clinical relevance of the presence of LC findings at the site of the DH has not yet been determined. We conducted a prospective study enrolling a total of 98 glaucoma eyes with DH and 37 OAG eyes with focal LC defect without DH to determine whether visual field (VF) progression differs according to the findings of the LC that had been evaluated by enhanced depth imaging (EDI) of optical coherence tomography (OCT) and its relationship with DH. Only the presence of focal LC defects was significantly different between the progressing and stable patients (*P* < 0.001). Baseline intraocular pressure (hazard ratio [HR], 1.076; *P* = 0.098) and the presence of focal LC defects at the DH site (HR, 2.620; *P* = 0.002) were found to be associated with VF progression. Glaucoma eyes with DH at the site of focal LC defects showed frequent and faster VF progression compared with DH not accompanied by LC alterations or LC alterations not accompanied by DH. Evaluating LC alterations in glaucoma eyes with DH may be important in predicting the progression of glaucoma.

## Introduction

Disc hemorrhage (DH) is a significant risk factor for the development and progression of glaucoma^1, 2^, this was further confirmed in the Ocular Hypertension Treatment Study^3^. In the Early Manifest Glaucoma Trial and Collaborative Normal-Tension Glaucoma Study, DH was significantly associated with glaucoma progression^1, 4^. With the use of spectral-domain optical coherence tomography (SD-OCT) with enhanced depth imaging (EDI) or swept-source OCT with longer wavelengths, it is now possible to obtain images of the lamina cribrosa (LC) *in vivo*. Several OCT studies reported LC alterations in glaucomatous eyes with DH, which included focal LC defects and laminar disinsertions^5–8^. LC changes at the site of DH have been proposed as a cause of DH development in eyes with glaucoma^9^. Nevertheless, these LC findings are not found in all cases of DH using OCT. Chauhan *et al*. reported that although laminar disinsertions were found twice as frequently in eyes with DH, only 39% of DHs were located within a laminar disinsertion^5^. Similarly, Park *et al*.^7^ reported that focal LC defects that corresponded to the location of DHs were found in only 62.1% of glaucomatous eyes. Moreover, the clinical relevance of the presence of LC findings at the site of the DH has not yet been determined.

In this study, we prospectively enrolled open-angle glaucoma (OAG) patients with DH and analyzed patients who were followed-up for at least 4 years. Patients were classified according to their LC findings, as being with or without focal LC defects at the location of the DH. The clinical characteristics and visual field (VF) progression in OAG eyes with a DH according to the findings of the LC were compared to determine the clinical relevance of focal LC defects at the site of the DH in OAG eyes. To be ensure, we also compared the progression of OAG eyes with focal LC defects associated with DH to eyes with focal LC defects without DH.

## Results

A total of 147 eyes, of 147 OAG patients with DH who met the inclusion and exclusion criteria, were enrolled in this study. Of these 147 eyes, 12 (8.2%) were excluded from further analysis due to poor OCT scan images of the LC or inability to identify a clear contour of the LC. The remaining 135 eyes were analyzed. Baseline characteristics are listed in Table 1. All patients were under glaucoma medication, which 78 (57.8%) used prostaglandins. Glaucoma patients with DH had the following characteristics. The mean age was 60.88 ± 12.09 years, and the mean spherical equivalent (SE) refractive error was −1.18 ± 2.99 D. The mean number of DHs found during the follow-up period was 2.13 ± 1.09 in all patients (range, 1–4). There were 62 (63.3%) eyes with recurrent DH. Focal LC defects on the EDI OCT images were found in 36 (36.7%) eyes at baseline. Among these 36 eyes with focal LC defects, 20 (55.6%) eyes had a focal LC defect at the DH site and 16 (44.4%) had a focal LC defect outside the DH site. Glaucoma patients with focal LC defect without DH had similar baseline characteristics compared to eyes with DH (Table 1).

**Table 1:** Demographics and clinical characteristics of glaucoma patients with disc hemorrhage.

Variables	Glaucoma patients with DH	Glaucoma patients with focal LC defect without DH	*P* Value
Patients (no.)	98	37	
Age at diagnosis (yrs)	60.88 ± 12.09	60.05 ± 12.32	0.921^*^
Male, no. (%)	36 (36.7%)	8 (21.6%)	0.104^†^
Systemic factors, no (%)			
Diabetes mellitus	18 (18.4%)	5 (13.5%)	0.613^†^
Hypertension	25 (25.5%)	6 (16.2%)	0.181^†^
Medication of aspirin	24 (24.5%)	8 (21.6%)	0.823^†^
SE refraction error (D)	−1.18 ± 2.99	−1.63 ± 2.63	0.540^*^
Axial length (mm)	24.16 ± 1.66	24.26 ± 1.49	0.775^*^
Central corneal thickness (μm)	526.43 ± 35.01	524.86 ± 31.84	0.115^*^
Baseline IOP (mmHg)	15.24 ± 3.33	16.35 ± 3.00	0.604^*^
Mean follow-up IOP (mmHg)	14.30 ± 2.63	14.02 ± 3.70	0.138^*^
Peak follow-up IOP (mmHg)	15.70 ± 3.21	15.62 ± 3.34	0.974^*^
IOP fluctuation (mmHg)	4.27 ± 1.61	4.62 ± 1.01	0.932^*^
Ocular characteristics			
Disc area (mm^2^)	2.17 ± 0.45	2.16 ± 0.44	0.620^*^
Average RNFL thickness (μm)	79.91 ± 11.69	81.51 ± 10.60	0.467^*^
Number of DH	2.13 ± 1.09	0	
Recurrent DH, no (%)	62 (63.3%)	0	
Number of eyes with focal LC defect, no (%)	36 (36.7%)	37 (100%)	
Focal LC defect at the DH site, no (%)	20 (55.6%)		
Focal LC defect outside the DH site, no (%)	16 (44.4%)		
Baseline VF examination			
MD (dB)	−3.98 ± 4.82	−3.54 ± 3.10	0.612^*^
PSD (dB)	4.59 ± 3.77	4.65 ± 3.06	0.928^*^
Follow-up duration (mos)	59.48 ± 9.21	58.67 ± 9.65	0.470^*^
Number of VF examination (no.)	7.02 ± 2.24	6.47 ± 1.21	0.526^*^

D, diopters; IOP, intraocular pressure; RNFL, retinal nerve fiber layer; DH, disc hemorrhage; LC, lamina cribrosa; MD, mean deviation; PSD, pattern standard deviation; VF, visual field.

Data are mean ± standard deviation unless otherwise indicated.

^*^Student t-test.

^†^Chi-square test.

Data are mean ± standard deviation unless otherwise indicated.

Overall, there was a mean of 7.02 ± 2.24 VF examinations per eye, and VF progression was detected in 45 eyes (45.9%) during a mean follow-up of 59.48 ± 9.21 months in eyes with DH. The estimated slope for MD was −0.35 ± 0.93 dB/year in all patients, which was calculated using a linear mixed effects model. Comparison between the progressing patients with DH and stable patients with DH are shown in Table 2. Among the progressing patients with DH, 26 (57.8%) had focal LC defects at baseline. Among the stable patients with DH, 10 (18.9%) had focal LC defects. Only the presence of a focal LC defect was significantly different between the progressing and stable patients (*P* < 0.001). The frequency of focal LC defects at the DH site was significantly different between the progressing and stable patients (*P* = 0.001), but there was no group difference in the frequency of focal LC defects outside the DH site (*P* = 0.263). Parameters for VF progression using the Cox proportional hazard model are shown in Table 3. Baseline IOP (HR, 1.076; 95% CI, 0.899–1.170; *P* = 0.098) and the presence of a focal LC defect at the DH site (HR, 2.620; 95% CI, 1.421–4.831; *P* = 0.002) were each found to be associated with VF progression (*P* < 0.10) in univariate analyses. Multivariate analyses also showed that a focal LC defect at the DH site (HR, 2.502; 95% CI, 1.265–4.948; *P* = 0.008) was significantly related to VF progression in OAG patients with DH (Table 4).

**Table 2:** Comparison between glaucoma patients with disc hemorrhage who showed visual field progression or remained stable during the follow-up period.

Variables	Progression (n = 45)	Stable (n = 53)	*P* Value
Age at diagnosis (yrs)	61.62 ± 12.21	60.26 ± 12.07	0.582^*^
Male, no. (%)	22 (41.5%)	14 (31.1%)	0.197^†^
Systemic factors, no (%)			
Diabetes mellitus	8 (17.8%)	10 (18.9%)	0.551^†^
Hypertension	11 (24.4%)	14 (26.4%)	0.505^†^
Medication of aspirin	11 (24.4%)	13 (24.5%)	0.590^†^
SE refraction error (D)	−1.51 ± 3.19	−0.78 ± 2.75	0.389^*^
Axial length (mm)	24.20 ± 1.47	24.10 ± 1.87	0.846^*^
Central corneal thickness (μm)	519.05 ± 39.21	531.62 ± 31.45	0.234^*^
Baseline IOP (mmHg)	14.82 ± 3.57	15.55 ± 3.14	0.337^*^
Mean follow-up IOP (mmHg)	14.04 ± 2.92	14.52 ± 2.36	0.367^*^
Peak follow-up IOP (mmHg)	15.66 ± 3.35	15.75 ± 3.07	0.885^*^
IOP fluctuation (mmHg)	4.35 ± 1.63	4.17 ± 4.59	0.583^*^
Optic disc characteristics			
Disc area (mm^2^)	2.20 ± 0.51	2.14 ± 0.38	0.547^*^
Average RNFL thickness (μm)	79.82 ± 12.74	79.97 ± 10.84	0.950^*^
Number of DH	2.15 ± 1.10	2.11 ± 1.08	0.849^*^
Recurrent DH, no (%)	29 (64.4%)	33 (62.3%)	0.495^*^
Number of eyes with focal LC defect, no (%)	26 (57.8%)	10 (18.9%)	<**0.001** ^**†**^
Focal LC defect at the DH site, no (%)	17 (37.8%)	3 (5.7%)	**0.001** ^**†**^
Focal LC defect outside the DH site, no (%)	9 (20.0%)	7 (13.2%)	0.263
Baseline VF examination	−4.22 ± 3.69	−3.77 ± 4.91	0.654^*^
MD (dB)	4.31 ± 3.69	4.84 ± 3.86	0.499^*^
PSD (dB)	72.29 ± 12.24	72.64 ± 12.16	0.161^*^
Follow-up duration (mos)			

D, diopters; IOP, intraocular pressure; RNFL, retinal nerve fiber layer; DH, disc hemorrhage; LC, lamina cribrosa; MD, mean deviation; PSD, pattern standard deviation; VF, visual field.

^*^Student t-test.

^†^Chi-square test.

Data are mean ± standard deviation unless otherwise indicated.

Factors with statistical significance are shown in bold.

**Table 3:** Cox proportional hazards univariate analysis of visual field progression in glaucoma patients with disc hemorrhage.

Variables	Univariate HR (95% CI)	*P* Value
Age at diagnosis (for each year older)	1.013 (0.987–1.039)	0.342
Male gender	1.345 (0.710–2.549)	0.363
Diabetes mellitus	1.357 (0.626–2.940)	0.440
Hypertension	1.076 (0.542–2.135)	0.835
Medication of aspirin	1.245 (0.625–2.480)	0.534
SE refraction error (D)	1.005 (0.889–1.137)	0.931
Axial length (mm)	0.896 (0.666–1.204)	0.896
Central corneal thickness (μm)	1.001 (0.990–1.012)	0.871
Baseline IOP (mmHg)	1.076 (0.889–1.170)	**0.098**
Mean follow-up IOP (mmHg)	1.089 (0.976–1.214)	0.859
Peak follow-up IOP (mmHg)	1.010 (0.919–1.109)	0.836
IOP fluctuation (mmHg)	0.981 (0.814–1.182)	0.837
Disc area (mm^2^)	1.072 (0.527–2.179)	0.848
Average RNFL thickness (μm)	0.993 (0.968–1.020)	0.621
Number of DH detection	1.001 (0.990–1.026)	0.342
DH recurrence	0.809 (0.436–1.502)	0.502
Focal LC defect at the DH site	2.620 (1.421–4.831)	**0.002**
Baseline MD (for each dB worse)	1.069 (0.966–1.136)	0.353
Follow-up duration (mos)	0.874 (0.671–1.137)	0.314

CI, confidence interval; HR, hazard ratio; D, diopters; IOP, intraocular pressure; RNFL, retinal nerve fiber layer; DH, disc hemorrhage; LC, lamina cribrosa; MD, mean deviation; PSD, pattern standard deviation; VF, visual field.

Variables with P < 0.10 are shown in bold.

**Table 4:** Cox proportional hazards multivariate analysis of visual field progression in glaucoma patients with disc hemorrhage.

Variables	Multivariate HR (95% CI)	*P* Value
Baseline IOP	1.053 (0.857–1.116)	0.855
Focal LC defect at the DH site	2.502 (1.265–4.948)	**0.008**

CI, confidence interval; HR, hazard ratio; IOP, intraocular pressure; DH, disc hemorrhage; LC, lamina cribrosa.

Variables with P < 0.10 in the univariate model were entered in a multivariate model.

Factors with statistical significance are shown in bold.

**Figure 1:**
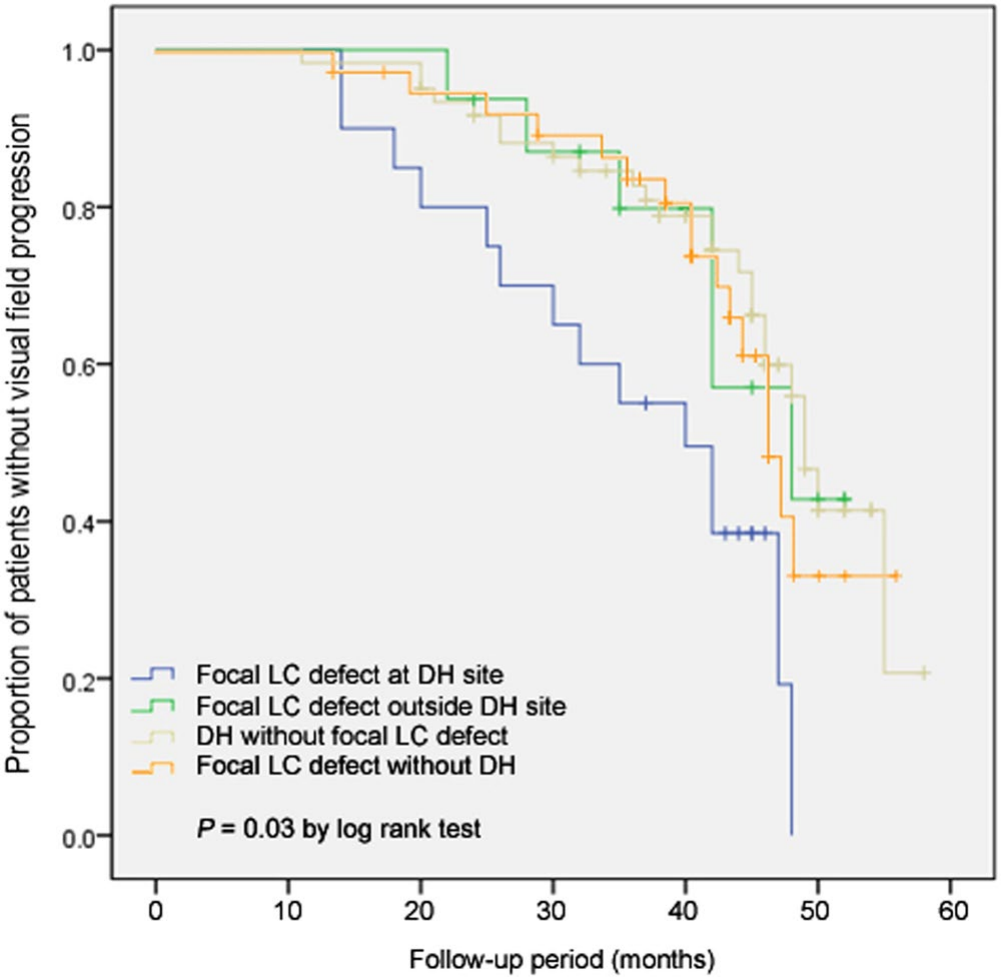
Kaplan-Meier survival curve for visual field progression in glaucoma eyes with disc hemorrhage according to the findings of the lamina cribrosa (LC) and eyes with focal LC defect without disc hemorrhage.

Further analyses were performed by comparing DH eyes with a focal LC defect at the DH site (n = 20), DH eyes with a focal LC defect outside the DH site (n = 16), DH eyes without a focal LC defect (n = 62), and eyes with focal LC defect without DH (n = 37). Among them, 14 (70.0%) DH eyes with a focal LC defect at the DH site, 6 (37.5%) DH eyes with a focal LC defect outside the DH site, 25 (40.3%) DH eyes without a focal LC defect, and 16 (43.2%) eyes with focal LC defect without DH showed VF progression; statistical significance were found between focal LC defect at the DH site and DH without focal LC defect (*P* = 0.009), between focal LC defect at DH site and focal LC defect without DH (*P* = 0.050), and between focal LC defect at the DH site and the rest groups (*P* = 0.033). The estimated slope for the MD was −0.96 ± 0.72 dB/year in DH eyes with a focal LC defect at the DH site, −0.27 ± 0.69 dB/year in DH eyes with a focal LC defect outside the DH site, −0.12 ± 1.11 dB/year in DH eyes without a focal LC defect, and −0.23 ± 0.47 dB/year in eyes with focal LC defect without DH (*P* = 0.046; Table 5). Kaplan-Meier survival analyses for VF progression showed a statistically significant difference in the cumulative probability of survival among the three groups, with lowest survival probability for the DH eyes with focal LC defect at the DH site (*P = *0.030; log-rank test) (Fig. 1).

**Table 5:** Comparison of visual field progression in glaucoma patients with disc hemorrhage according to the findings of the lamina cribrosa and its relationship with the disc hemorrhage location.

Variables	Focal LC defect at the DH site (n = 20)	Focal LC defect outside the DH site (n = 16)	DH without focal LC defect (n = 62)	Focal LC defect without DH (n = 37)	*P* Value
VF progression, no (%)	14 (70.0%)	6 (37.5%)	25 (40.3%)	16 (43.2%)	**0.112** ^*^
Focal LC defect at the DH site vs. Focal LC defect outside the DH site, *P* = 0.053					
Focal LC defect at the DH site vs. DH without Focal LC defect, *P* = 0.020					
Focal LC defect at the DH site vs. Focal LC defect without DH, *P* = 0.048					
Focal LC defect at the DH site vs. other three groups, *P* = 0.015					
MD slope (dB/yr)	−0.96 ± 0.72	−0.27 ± 0.69	−0.12 ± 1.11	−0.23 ± 0.47	**0.046** ^**†**^
Focal LC defect at the DH site vs. Focal LC defect outside the DH site, *P* = 0.057					
Focal LC defect at the DH site vs. DH without Focal LC defect, *P* = 0.009					
Focal LC defect at the DH site vs. Focal LC defect without DH, *P* = 0.050					
Focal LC defect at the DH site vs. other three groups, *P* = 0.033					

DH, disc hemorrhage; LC, lamina cribrosa; VF, visual field; MD, mean deviation.

^*^Chi-square test.

^†^Linear mixed-effect model.

Factors with statistical significance are shown in bold.

Cox proportional hazard analyses were also used for subgroups according to the presence of a focal LC defect and its relationship with the DH (Table 6). Among the DH eyes with an LC defect at the DH site, a greater baseline IOP (HR, 1.205; 95% CI, 1.003–1.819; *P* = 0.048) and the presence of a focal LC defect (HR, 3.244; 95% CI, 1.040–5.279; *P* = 0.001) were independent predictors of VF progression. Among the DH eyes with a LC defect outside the DH site, the number of DHs detected (HR, 2.704; 95% CI, 1.233–5.929; *P* = 0.013), use of aspirin (HR, 2.393; 95% CI, 1.460–3.021; *P* = 0.021), and presence of a focal LC defect (HR, 5.240; 95% CI, 1.401–7.93; *P* = 0.001) were predictive parameters for VF progression. In DH eyes without a LC defect, a worse baseline MD (HR, 0.946; 95% CI, 0.904–0.987; *P* = 0.031) was the only significant predictive factor for VF progression. In eyes with focal LC defect without DH, higher baseline IOP (HR, 1.051; 95% CI, 0.946–1.133; *P* = 0.023) and thinner average RNFL thickness (HR, 0.901; 95% CI, 0.823–0.986; *P* = 0.031) were significant predictive factor for VF progression.

**Table 6:** Cox proportional hazards univariate and multivariate analysis of visual field progression in glaucoma patients with disc hemorrhage according to the findings of the lamina cribrosa.

Variables	Univariate Analysis		Multivariate Analysis	
	HR (95% CI)	*P* Value	HR (95% CI)	*P* Value
**With focal LC defect at DH site**				
Baseline IOP	1.205 (1.015–1.431)	**0.043**	1.350 (1.003–1.819)	**0.048**
Mean IOP	1.084 (0.944–1.151)	0.163		
IOP fluctuation	1.171 (0.921–1.488)	0.197		
Baseline MD	0.777 (0.639–0.946)	**0.098**		
Focal LC defect	3.160 (1.036–5.210)	**0.002**	3.244 (1.040–5.279)	**0.001**
**With focal LC defect outside DH site**				
Age	1.117 (1.042–1.197)	**0.002**	1.133 (1.078–1.204)	**0.037**
Baseline IOP	1.084 (0.929–1.149)	0.149		
Mean IOP	1.082 (0.949–1.118)	**0.071**		
IOP fluctuation	1.261 (0.936–1.699)	0.128		
Number of DH detection	1.675 (1.002–2.800)	**0.049**	2.704 (1.233–5.929)	**0.013**
Medication of aspirin	2.715 (0.910–8.099)	**0.073**	2.393 (1.460–3.021)	**0.021**
Focal LC defect	4.401 (1.030–6.437)	**0.001**	5.240 (1.401–7.903)	**0.001**
**Without focal LC defect**				
Number of DH detection	1.730 (1.503–1.959)	**0.097**		
Average RNFL thickness	0.963 (0.925–1.003)	**0.071**		
Baseline MD	0.917 (0.841–0.999)	**0.048**	0.946 (0.904–0.987)	**0.031**
**Focal LC defect without DH**				
Average RNFL thickness	0.893 (0.822–0.971)	**0.008**	0.901 (0.823–0.986)	**0.023**
Baseline MD	0.809 (0.630–1.039)	**0.097**		
Baseline IOP	1.020 (0.941–1.148)	**0.112**	1.051 (0.946–1.133)	**0.078**

CI, confidence interval; HR, hazard ratio; D, diopters; IOP, intraocular pressure; RNFL, retinal nerve fiber layer; DH, disc hemorrhage; LC, lamina cribrosa; MD, mean deviation; PSD, pattern standard deviation; VF, visual field.

Variables with P < 0.10 are shown in bold (Univariate analysis).

Variables with P < 0.05 are shown in bold (Multivariate analysis).

## Discussion

We showed that OAG eyes with DH had accompanying focal LC defects at the DH site in 20 of 98 (20.4%) eyes, and up to 37.8% of the DH eyes showing progression had focal LC defects at the DH site. DH eyes with focal LC defects at the DH site showed a MD slope of −0.96 ± 0.72 dB/year and 70% of them showed significant VF progression. Focal LC defects at the DH location were therefore a significant predictive factor for future VF progression. Based on these results, imaging the LC in OAG eyes with DH, and looking for LC alterations at the DH site, may help to predict the progression and thus guide treatment for OAG eyes with DH.

**Figure 2:**
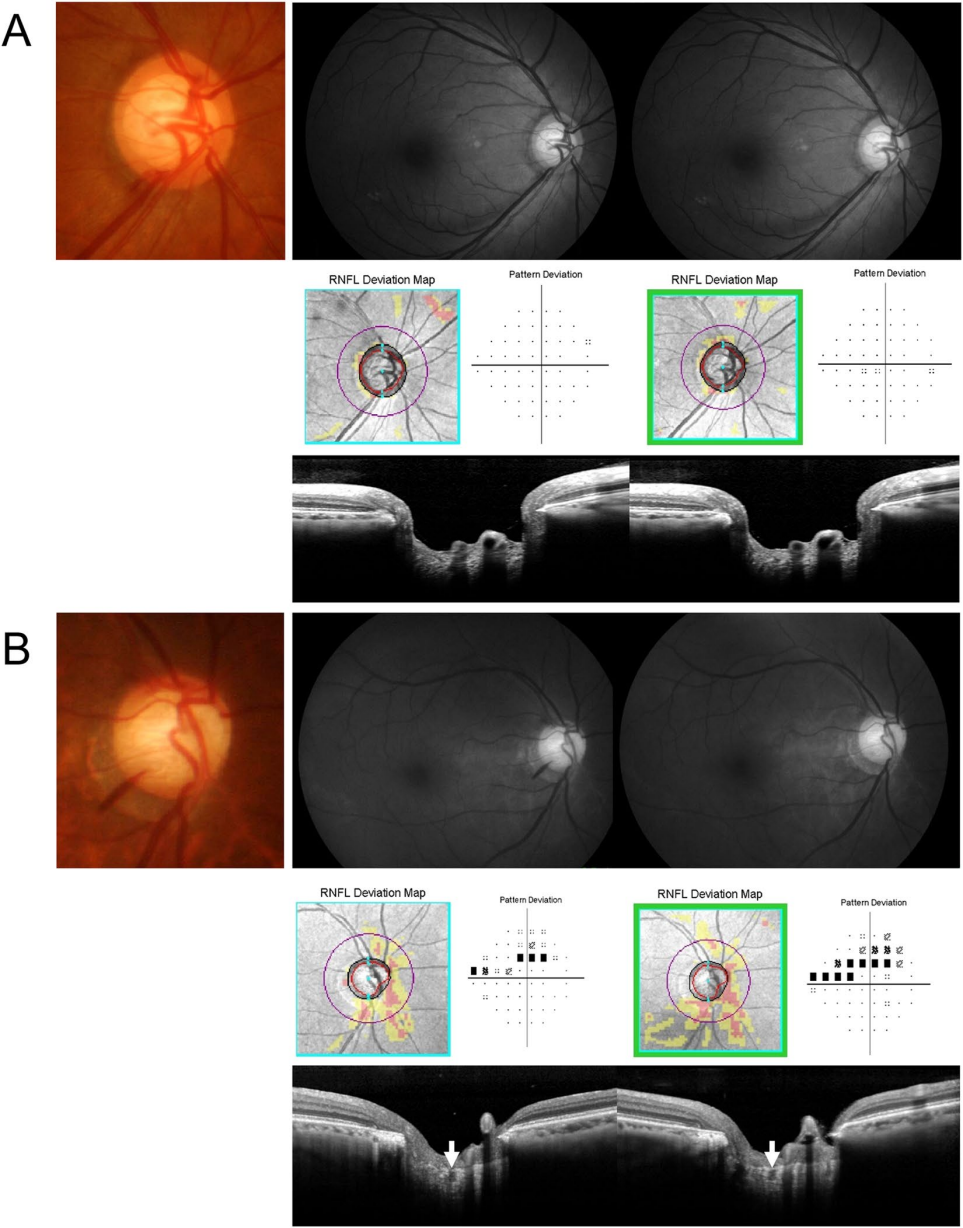
Representative cases. (**A**) A 56-year-old female with normal-tension glaucoma. A inferotemporal localized retinal nerve fiber layer (RNFL) defect with disc hemorrhage (DH) is shown in the right eye. Enhanced depth imaging (EDI) of the optic nerve head shows no focal lamina cribrosa (LC) defects. During five years of follow-up, there was no evidence of glaucoma progression in this case. (**B**) A 69-year-old male with normal-tension glaucoma. Diffuse RNFL defect with inferotemporal DH is shown in the right eye. Enhanced depth imaging (EDI) of the optic nerve head shows a focal lamina cribrosa (LC) defect at the site of DH. During four years of follow-up, there were progression in both the RNFL and the visual field.

Both DH and LC defects are signs of progressive glaucoma involving retinal nerve fiber layer (RNFL) defects and VF progression^10–13^. It has been suggested that there is a strong relationship between the location of the DH and the LC defect. Park *et al*. reported that DH was associated with focal LC defects in glaucoma patients^8^. Park *et al*. also reported that focal LC defects were found more frequently in glaucomatous eyes with DH compared to eyes without DH^7^. They reported that when the focal LC defect was found at the site of the DH, it was related to the DH area^6^. However, focal LC defects that were located within one-half clock-hour distance from the DH location were only found in approximately 56% of the eyes in this study. A recent study by Chauhan also reported that laminar disinsertions were frequently found in glaucomatous eyes with DH, which showed poor spatial concordance between the DH and the laminar disinsertion^5^. Only 39% of all DHs were located within a laminar disinsertion in their study. The results suggested that a spatial correlation between the DH and alterations in the LC may have a relationship that affects the prognosis of the DH as a risk factor for progression. We therefore characterized VF progression in OAG eyes with DH and analyzed the effect of focal LC defects. As shown in the Kaplan-Meier survival curves, OAG eyes with DH had a tendency to progress during the follow-up periods. However, when focal LC defects were present at the DH location, VF progression was faster in OAG eyes with DH at the DH site compared to eyes with focal LC defects outside the DH, or those without focal LC defects. As shown in the representative cases, OAG eye with DH without focal LC defects on the EDI ONH scans showed no glaucoma progression during follow-ups (Fig. 2A), however, eyes with DH accompanying focal LC defects showed VF and RNFL progression (Fig. 2B).

There have been few reports describing the risk factors for progression in glaucoma patients with DH. Previously, risk factors that were related to VF progression in glaucomatous eyes with DH were reported to include age and a worse baseline MD^14^. The results of our study showed that the related risk factors for future VF progression were different between DH eyes according to the LC findings. The risk factor for future VF progression in DH eyes without focal LC defects involved the baseline MD, showing that the baseline status is the most important parameter in these eyes. Our findings were consistent with a study by Liebmann, which reported that a worse VF status at baseline predicted further VF deterioration in glaucoma patients with DH^14^. However, in DH eyes with a focal LC defect not related to the DH location, or without a focal LC defect, several risk factors were found that suggested that vascular parameters were important, such as the number of DHs detected or the use of aspirin. An association between DH and vascular risk factors, including systemic hypertension, diabetes mellitus, and the use of aspirin, has been reported previously^15^. In contrast to these findings, in DH eyes with LC defects at the DH site, the baseline IOP and the presence of LC defects were significant risk factors for predicting future VF progression. These results suggest that mechanical stress resulting from the IOP may increase the risk of progression in DH eyes with focal LC defects at the site of the DH. When managing OAG patients with DH, LC findings may therefore provide information regarding treatment options and risk factors related to progression. Since focal LC defects are related to many important findings of glaucoma, such as localized RNFL defects, DH, and glaucoma progression, all these findings may be a clinical spectrum^8, 16, 17^. Therefore, focal LC defects accompanying other structural aspects that contribute to glaucoma progression including DH are important in managing these patients.

In previous reports, the change in the MD of DH eyes, which ranged from −0.17 ± 0.27 to −1.1 ± 1.3 dB/year, depended on the study^14, 18, 19^. In the present study, the global change in the VF showed a MD slope of −0.96 ± 0.72 dB/year in DH eyes with focal LC defects at the DH site. This was a faster rate of MD change compared to DH eyes with focal LC defects outside the DH site (−0.27 ± 0.69 dB/year) or DH eyes without a focal LC defect (−0.12 ± 1.11 dB/year). Liebmann *et al*. reported that localized progression corresponding to the DH location after the detection of DH was up to −3.7 ± 3.6 dB/year^20^. Eyes with focal LC defects have shown significant progression both globally and locally when compared with eyes without focal LC defects. Global VF progression was reported to be −0.54 dB/year in eyes with a focal LC defect, with a localized VF progression rate of −2.85 dB/year^13^. Both the DH and the focal LC defect therefore affect the localized VF progression, as shown in previous studies. Although the underlying developmental mechanism for DHs and focal LC defects are not well understood, our results suggest that when the two factors are combined, a mechanical mechanism may more strongly contribute to the progression of glaucoma.

Our study had some limitations. First, it had a relatively small sample size. There was relatively a small portion of patients with advanced glaucoma. We did not include glaucoma eyes without a DH. Further study is needed to confirm our findings in patients with advanced glaucoma. Second, it is difficult to generalize our findings because only Korean patients were studied. Since most of Korean glaucoma patients are normal-tension glaucoma (NTG) and our study subjects also had untreated IOP in the range of NTG, our finding could be considered as characteristics of NTG patients with DH. Third, the number and sites of DHs and focal LC defects may have been underestimated in some patients; we only included DHs that were detected. However, we mitigated the potential effects of this problem by including only patients who had been followed-up for at least 4 years, with photographs taken every 6–12 months. Fourth, there may have been issues regarding poor visualization of the LC under the optic disc rim and vessels. It is possible that some LC alterations in the areas with poor OCT penetration may have been missed. To reduce false-positive detection of focal LC defects, we defined a LC defect as having a diameter of ≥100 μm and a depth of >30 μm, per the study of Kiumehr *et al* ^17^. The LC defect also had to be present in two neighboring B-scans. The definition of LC defects was based on previous studies and may not be ideal. However, previous studies have stated that the definition we used may exclude normal anatomical variations and artifacts. Finally, the follow-up period was relatively short. Further investigation is needed to determine the long-term effects of DHs and focal LC defects on glaucoma progression.

In conclusion, we found that OAG eyes with DH at the site of a focal LC defect showed more frequent and faster VF progression compared to eyes with DH not accompanying LC alterations. Predictive risk factors for progression differed according to the LC findings in OAG eyes with DH. Therefore, evaluating LC alterations by scanning the ONH in OAG eyes with DH may be important in predicting and monitoring the progression of glaucoma.

## Methods

### Subjects

This study was based on the Glaucoma Progression Study conducted at Seoul St. Mary’s Hospital, Seoul, Republic of Korea, which has been ongoing since March 2009. The study was approved by the Institutional Review Board of Seoul St. Mary’s Hospital and the study protocol followed the tenets of the Declaration of Helsinki. Written informed consent was obtained from consecutive patients who met the eligibility criteria and were willing to participate in the study.

The database of patients included in this study from March 2012 was reviewed by two authors (H.Y.P and C.K.P). OAG patients with DH who had undergone at least six VF examinations, with follow-up for at least 4 years after the detection of the initial DH, were selected. For the initial work-up, each patient received a complete ophthalmic examination, including a review of their medical history, measurement of best-corrected visual acuity, refraction measurements, slit-lamp biomicroscopy, gonioscopy, Goldmann applanation tonometry, measurement of the central corneal thickness using ultrasound pachymetry (Tomey Corp., Nagoya, Japan), measurement of axial length using ocular biometry (IOL Master; Carl Zeiss Meditec, Dublin, CA, USA), dilated stereoscopic examination of the optic disc, disc and red-free fundus photography (Canon, Tokyo, Japan), Heidelberg retina tomography (HRT; Heidelberg Engineering, Heidelberg, Germany), Cirrus OCT (Carl Zeiss Meditec), and Humphrey VF examination using the Swedish interactive threshold algorithm Standard 24–2 test (Carl Zeiss Meditec). The EDI scanning of the optic nerve head (ONH) using the Spectralis OCT (Heidelberg Engineering) was performed at baseline and 12 months thereafter. When a DH was detected, additional EDI scanning using the Spectralis OCT was performed. Color disc and fundus photography, VF, and OCT examinations were repeated at 6-month intervals during the first 3 years after the glaucoma diagnosis, and then at 12 months thereafter.

Patients had to meet the following criteria for a diagnosis of glaucoma: glaucomatous optic disc appearance (such as diffuse or localized rim thinning, a notch in the rim, or a vertical cup-to-disc ratio greater than that of the other eye by 0.2); a VF consistent with glaucoma (a cluster of ≥3 non-edge points on a pattern deviation plot with a probability of <5% of the normal population, with one of these points having a probability of <1%), a pattern standard deviation with a *P*-value < 5% or a Glaucoma Hemifield Test result outside the normal limits in a consistent pattern on two qualifying VFs, confirmed by two glaucoma specialists (H.Y.P. and C.K.P.), and an open-angle on gonioscopy.

All patients had to meet the following additional inclusion criteria for inclusion in the study: a best-corrected visual acuity ≥20/40; a spherical refraction within ±6.0 diopters (D); a cylinder correction within ±3.0 D; consistently more than six reliable VFs (defined as a false negative <15%, a false positive <15%, and fixation losses <20%); and a mean deviation (MD) better than −20.00 decibels (dB). Patients were excluded on the basis of any of the following criteria: a history of any retinal disease, including diabetic or hypertensive retinopathy; a history of eye trauma or surgery with the exception of uncomplicated cataract surgery; other optic nerve disease besides glaucoma; and a history of systemic or neurological diseases that might affect the VF. If glaucoma incisional or laser treatment was performed during the follow-up, only the data obtained in the period before the treatment were analyzed. If both eyes of a glaucoma patient had DH and met the inclusion and exclusion criteria, one eye was randomly chosen for the study.

DH was defined as an isolated flame-shape or splinter-like hemorrhage on the optic disc or peripapillary area extending to the optic disc border. Alternative causes of hemorrhage were excluded by diagnostic testing for ischemic optic neuropathy, papillitis, retinal vein occlusion, diabetic retinopathy, and posterior vitreous detachment. All DHs that occurred during the follow-up were recorded. The clock-hour location of the DH was recorded according to its proximal location. Some eyes had a recurrent DH (>1 DH during the follow-up). The intraocular pressure (IOP) was recorded at each visit. Baseline untreated IOP was the IOP at the initial visit with no glaucoma medication. The mean IOP during the entire follow-up period was calculated by averaging all measurements. The IOP fluctuation was defined as the standard deviation of all measurements.

### EDI-OCT

Serial horizontal and vertical cross-sectional scans covering the ONH, approximately 30 μm apart, were obtained using the EDI technique of the Spectralis OCT for LC analyses, as described previously^21–23^. Each section incorporated an average of at least 35 OCT frames. Images had a quality score >15, with approximately 65–70 sections per eye. Volume scans, covering 9.0 mm × 6.0 mm in the macular area centered on the fovea, using the Spectralis OCT involving 25 sections, were performed on each eye. The EDI OCT images of the ONH at baseline were reviewed by an experienced glaucoma specialist (Y.J.) who was blinded to the clinical information of the patients. The examination focused on the presence of any alteration in the smooth curvilinear U- or W-shaped cross-sectional contour of the LC with upward sloping at the far periphery of the LC toward its insertion^13–17^. Alterations of the LC were defined using the guidelines specified by Kiumehr *et al*.^17^, and focal LC defects were observed in the study. A focal LC defect was defined as a hole-like defect with a discontinuous anterior laminar surface that appeared to be a full thickness defect. The focal LC defect had to be present in two neighboring B-scans to avoid false positives in both the horizontal and vertical scans. Eyes with more than one focal LC defect were excluded.

Based on the LC findings, patients with DH were divided into three groups according to the DH; DH eyes with a LC defect at the site of the DH, DH eyes with a LC defect outside the site of the DH, and DH eyes without a LC defect. Focal LC defects within the clock-hour where the proximal location of the DH was located were regarded as an LC defect at the site of the DH.

### VF Analysis and Definition of VF Progression

The first VF test was excluded to avoid any effects of the learning process. The second VF examination was performed within 1 month of the first visit. The average values from the first two reliable fields were used for the baseline MD value. Follow-up VF tests were analyzed based on the Early Manifest Glaucoma Trial criteria that defined VF progression^24^. The average of two baseline field measurements was compared with those of subsequent tests using glaucoma change probability maps, based on the pattern deviation. Progression was defined to have occurred if there was statistically significant deterioration (*P < *0.05) in at least three locations on pattern deviation change probability maps that did not have to be contiguous. This significant deterioration was confirmed using two consecutive tests. To calculate the VF progression rate, the time course of MD was evaluated.

### Statistical Analysis

Student’s *t*-test was used to compare differences between groups. The *chi*-squared test was used where appropriate to compare frequencies. Univariate and multivariate Cox proportional hazard ratios (HRs) were used with a 95% confidence interval (CI) calculated using univariate and multivariate models to identify risk factors predictive of VF progression. Variables with *P* < 0.10 in the univariate model were entered in a multivariate model. Kaplan-Meier survival curves were created to compare the survival times for eyes with and without LC defects. The first time that functional deterioration was found was regarded as the end point in survival analyses. A *P* value < 0.05 was considered statistically significant.

To calculate the VF progression rate, the MD slope was determined by linear regression analyses using the MD value. Because the data from the same patient were correlated with each other, linear mixed-effects model analyses were used. The regression coefficient of the time course of MD was determined, and the progression rates were compared between the two groups.
